# Supplementary material for: The interactome of intact mitochondria by cross-linking mass spectrometry provides evidence for coexisting respiratory supercomplexes
Source: Mol Cell Proteomics. 2017 Dec 8;17(2):216–32. doi: 10.1074/mcp.RA117.000470 (PMC5795388; doi:10.1074/mcp.RA117.000470)
Supplement: Supplemental Data [file supp_RA117.000470_133922_0_supp_23978_fzffwf.zip › spectra_annotation/mito_DR_spectra_annotation/458-1-15-1-14-1.pdf]

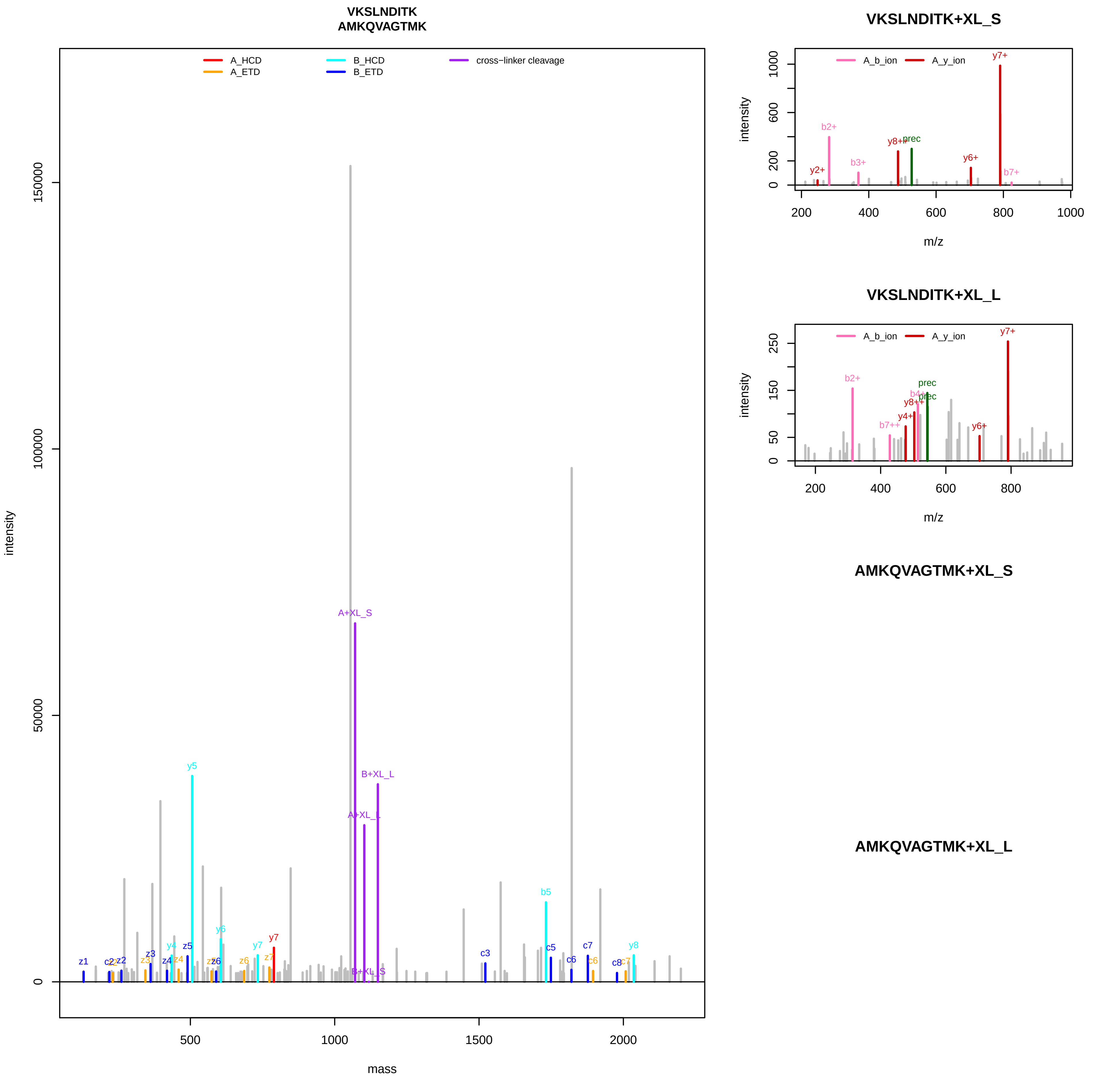
VKSLNDITK
AMKQVAGTMK
A_HCD
A_ETD
B_HCD
B_ETD
cross−linker cleavage
A+XL_S
B+XL_L
A+XL_L
B+XL_S
y5
y6
y7
y8
b5
z1
c2
z2
z3
z4
z5
z6
z7
c3
c5
c6
c7
c8
intensity
mass
0
50000
100000
150000
500
1000
1500
2000
VKSLNDITK+XL_S
A_b_ion
A_y_ion
b2+
b3+
b7+
y2+
y6+
y7+
y8++
prec
m/z
VKSLNDITK+XL_L
b4+
b7++
y4+
AMKQVAGTMK+XL_S
AMKQVAGTMK+XL_L
